# Development and usage of an anesthesia data warehouse: lessons learnt from a 10-year project

**DOI:** 10.1007/s10877-022-00898-y

**Published:** 2022-08-06

**Authors:** Antoine Lamer, Mouhamed Djahoum Moussa, Romaric Marcilly, Régis Logier, Benoit Vallet, Benoît Tavernier

**Affiliations:** 1 Univ. Lille, CHU Lille, ULR 2694 - METRICS: Évaluation des Technologies de Santé et des Pratiques Médicales, Lille, France; 2 InterHop, Rennes, France; 3 Univ. Lille, CHU Lille, Cardiovascular Anesthesia and Intensive Care, Lille, France; 4 CHU Lille, CIC-IT 1403 - Investigation Center, Lille, France; 5 CHU Lille, Pôle d’Anesthésie-Réanimation, 59000 Lille, France

**Keywords:** Anesthesia information management system, Data warehouse, Data reuse, Database, Retrospective studies

## Abstract

This paper describes the development and implementation of an anesthesia data warehouse in the Lille University Hospital. We share the lessons learned from a ten-year project and provide guidance for the implementation of such a project. Our clinical data warehouse is mainly fed with data collected by the anesthesia information management system and hospital discharge reports. The data warehouse stores historical and accurate data with an accuracy level of the day for administrative data, and of the second for monitoring data. Datamarts complete the architecture and provide secondary computed data and indicators, in order to execute queries faster and easily. Between 2010 and 2021, 636 784 anesthesia records were integrated for 353 152 patients. We reported the main concerns and barriers during the development of this project and we provided 8 tips to handle them. We have implemented our data warehouse into the OMOP common data model as a complementary downstream data model. The next step of the project will be to disseminate the use of the OMOP data model for anesthesia and critical care, and drive the trend towards federated learning to enhance collaborations and multicenter studies.

## Introduction

The Anesthesia Information Management System (AIMS) has gradually replaced the paper records in the last decades [1, 2]. This electronic version of the anesthesia record captures data automatically from clinical monitors and facilitates the documentation of the anesthesia procedure such as the main stages of the surgical intervention and anesthesia management. The benefits of an AIMS include improved readability of the anesthesia record and greater efficiency in documentation efforts [3].

Anesthesia records are mainly used for reviewing single patient record but also help searching, querying and retrieving data from thousands of cases to conduct retrospective analyses for clinical research or evaluate compliance with guidelines [4–6]. The main characteristics of the data recorded in the operating room are their sampling frequency and accuracy, with one measurement every 30 s for signals such as heart rate or invasive blood pressure. This very fine data is often transformed into new variables and more workable information. For example, the arterial pressure signal is computed into comprehensive hypotension events, such as the time below or beyond a threshold, the number of episodes, or the area under the curve [7]. Thus, the reuse of data collected automatically by AIMS has allowed to conduct several studies with thousands of interventions and to identify links between haemodynamic variations (e.g. hypotension) and negative post-operative outcomes (e.g. death, acute kindey injury) [8–11].

Reusing AIMS data faces several challenges. First of all, the data produced by the software are stored in a secured proprietary system, where it is impossible to add or modify the structure and the data. Secondly, even though AIMS provides analytical functions to count procedures or patients based on simple criteria (e.g. date, demographics or care units characteristics), the computation of hypotension involves more complex algorithms and larger volumes of data which could be time-consuming and slow down the performance of the software in its current use. In order to overcome the problems mentioned previously, a data warehouse is created in combining multiple operational databases and acts as a single and consolidated reference data to produce statistics and reports.. For this, an Extract-Transform-Load (ETL) process extracts all relevant data from operational databases, applies cleaning and transformation rules, and finally loads it into the data warehouse. Data warehouses were initially implemented by industry to assist in decision-making [12], and over the past 20 years, hospitals have also adopted this technology to facilitate the reuse of administrative and clinical data for research purposes [13–16].

This article describes the development and implementation of an anesthesia data warehouse in the Lille University Hospital. We share the lessons learned from a ten-year project and provide guidance for the implementation of such a project.

## Data warehouse implementation

The development of the data warehouse began in 2011 and lasted 4 years. We describe the design of the data warehouse, the data sources, the implementation process and the technologies we used for that. We illustrate the usages we had through data extraction for studies or dashboards.

### Design and data model

The need for the warehouse arose from an analysis of the recurring needs expressed by physicians. They highlighted the need for indicators derived from haemodynamic and ventilation signals—hypotension, hypertension, bradycardia, tachycardia, oxygen desaturation, low tidal volume. In addition to the details of the operating room period, it was necessary to integrate data on the patient's outcome, such as admission to intensive care, length of stay, mortality, organ support, biology results, and transfusions. These additional data come from other software, developed by different editors, and with each time, a database structure that is specific to the software. A concrete analytic need was to be able to compare the duration of hypotension across multiple thresholds with in-hospital mortality. A data warehouse would thus allow the pooling of data from these different data sources, and the calculation of new variables, without interfering with the routine use of source software.

We have chosen the architecture proposed by Inmon, in which the data warehouse is a “subject-oriented, nonvolatile, integrated, time-variant collection of data in support of management’s decisions.” [17]. Following this architecture, the data warehouse acts as a unified source where all data is integrated. It is modeled with a normalized data model, and stores historical and precise data. Thus, the data warehouse stores all the data, with their initial level of granularity. Depending on the source, the data are accurate to the day for hospital stay data (passage in the care units, medical acts), and to the second for operating room data (monitoring, stages of the intervention and administration of medication).

Based on the data warehouse, datamarts are then constituted with secondary computed data (cf. Feature Extraction section) and organized to facilitate queries. For this, the datamarts are denormalized (i.e. some data may be redundant). In the case of a clinical data warehouse, mainly developed for research purposes, this approach keeps the data warehouse as the primary element, with the patient as the central unit. We prefer this approach to the one proposed by Kimball, a bottom-up approach in which datamarts are first designed based on the business requirements. With this architecture, the datamarts are the central elements, in particular to produce data cubes for reporting purposes. By developing the datamarts in a second time only, the Kimball’s approach may miss some important data.

**Fig. 1:**
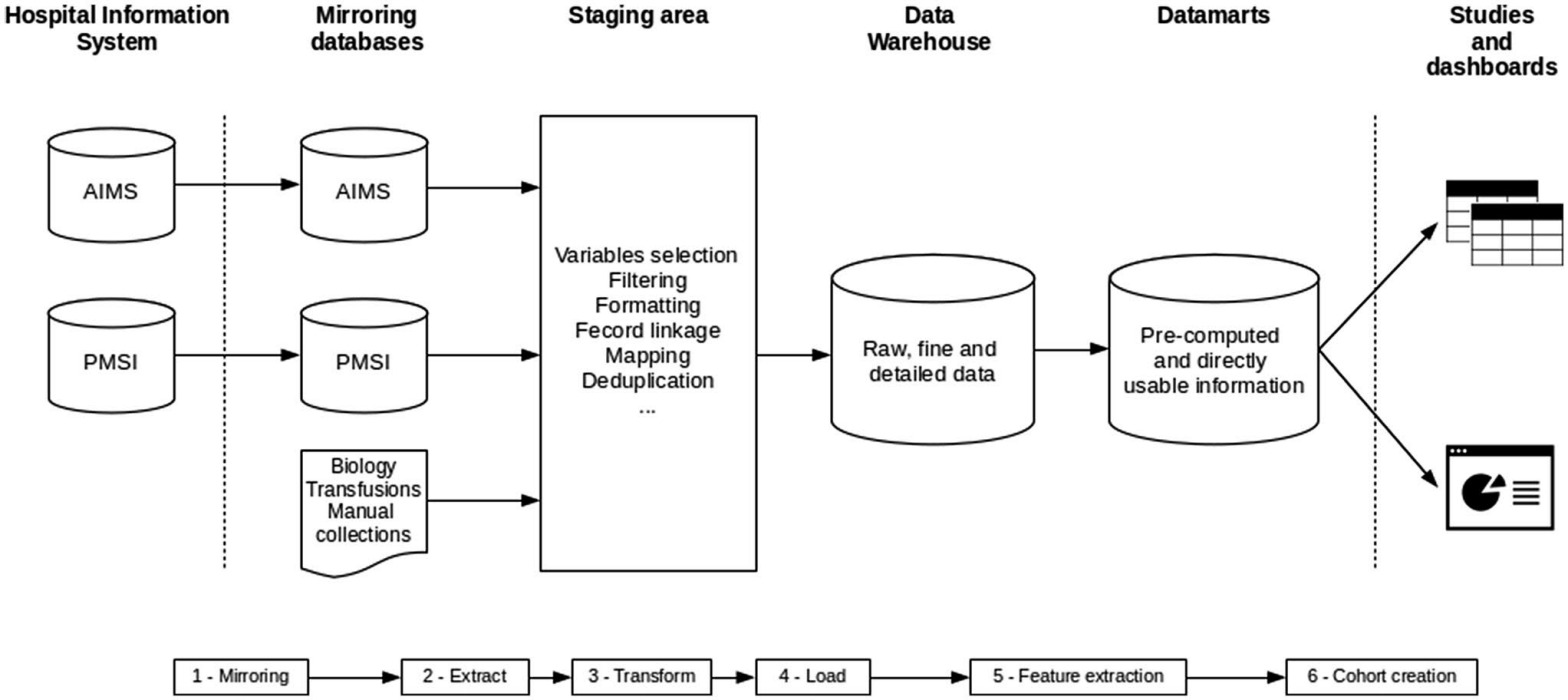
Data warehouse architecture and process. *AIMS* Anesthesia Information Management System, *PMSI* Programme de Médicalisation des Systèmes d’Information (hospital discharge reports)

**Fig. 2:**
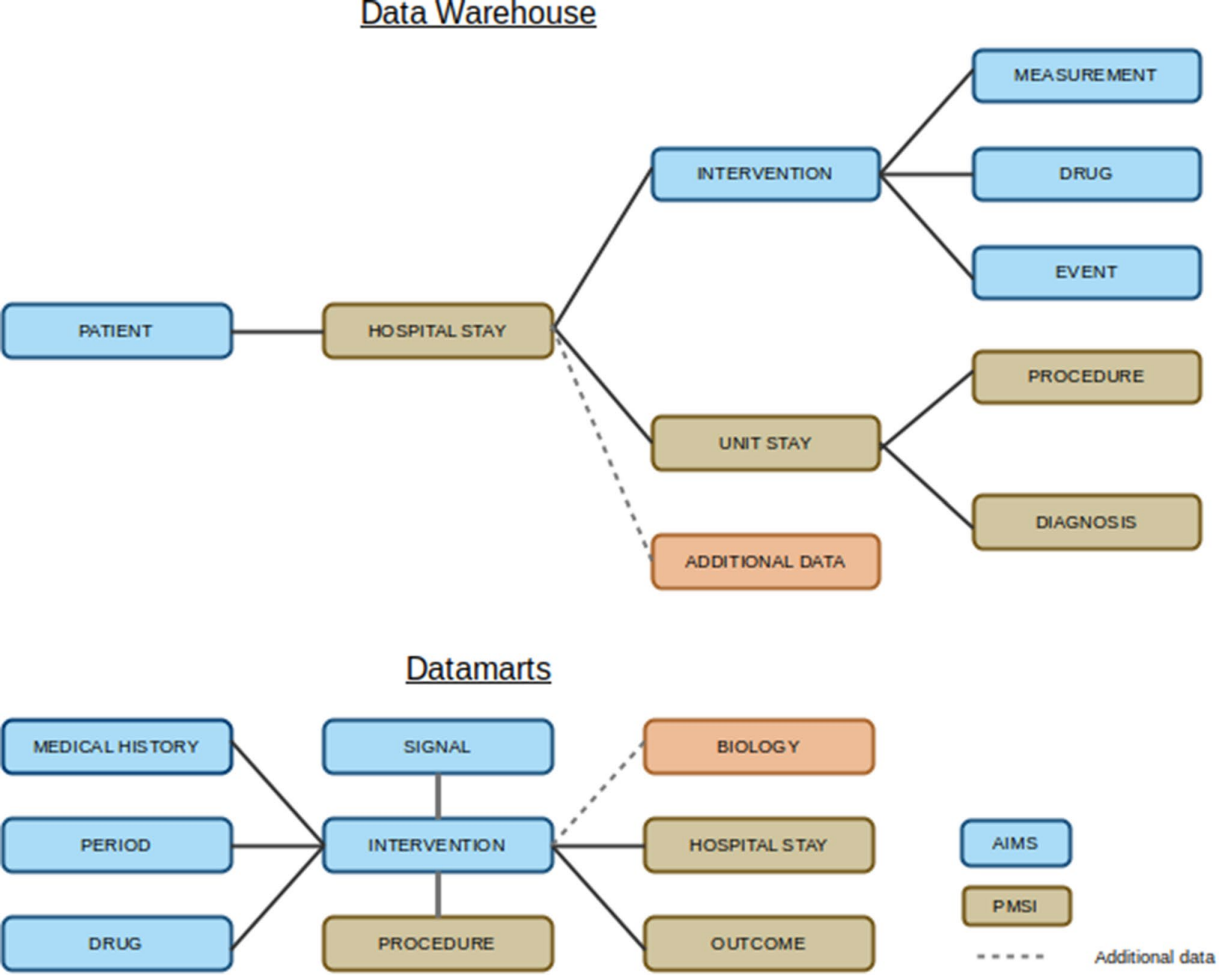
Data model of the data warehouse and the datamarts. Tables of the data warehouse store historical and precise data, at the greatest level of detail, and are linked to each other by foreign key relationships. Tables of the datamarts are constituted of secondary computed data

The architecture of the data warehouse and the loading process is described in Fig. 1 and fully detailed in the sections below. The data models of both data warehouse and datamarts are detailed in Fig. 2.

### Data sources

The main source of the data warehouse was the AIMS, DIANE ANESTHESIA (Bow Medical, Amiens, France) [18]. This software is exclusively dedicated to the documentation of anesthesia procedures. Different modules collect and centralize all data referring to one case, from pre-anesthetic evaluation to discharge from post-anesthesia care unit (PACU). The data collected includes patient demographics and history, continuous monitored parameters (e.g. heart rate, blood pressure, respiratory rate, tidal volume), drugs administrations and main steps of anesthesia and surgery procedures.

Intraoperative data were supplemented by hospital discharge reports from the *Programme de Médicalisation des Systèmes d’Information* (PMSI). These reports include all hospital stay characteristics, such as diagnoses [based on the French edition of the International Classification of Diseases 10th edition (ICD10)], medical procedures [based on the French terminology *Classification Commune des Actes Médicaux* (CCAM)], type of medical unit (e.g. intensive care), admission and discharge dates, status discharge (i.e. home, transfer or death).

In addition to these two data sources, other data were also integrated into the data warehouse. For example, we added biology results (e.g. creatinine) or transfusion data from two other hospital software in order to assess the impact of hypotension on renal function. Since 2019, the French National Institute of Statistics and Economic Studies (INSEE—« *Institut National de la statistique et des études économiques*») is delivering the French National Mortality Database (FNMD—« *fichier des personnes décédées»*) on a monthly basis. It contains tens of millions of records in open-access. Therefore, we have recently been able to add the out-of-hospital mortality.

The hospital information system is not yet fully computerized and some information is still collected in paper format (e.g. patient follow-up that has no impact on billing). In addition, some software does not provide automatic extraction solution. Therefore, manual collection may still be required to add this data to the data warehouse. Because of the time-consuming aspect, this solution is only feasible for a limited number of records.

### Vocabularies

Integrated records from the PMSI were annotated with national terminologies, i.e. the CIM10 for the diagnoses, and the CCAM for the medical procedures. We kept the local terminologies for the other vocabularies (i.e. measurements, units, drugs) such as defined in the AIMS. Some cleaning operations were applied to AIMS vocabularies when they were misspelled, or duplicated. It could occur that users entered items a second time without checking if they already existed, or that clinical monitors labeled items differently according to their brand (e.g. General Electrics or Mindray). Another cleaning operation consisted of combining elements of vocabularies (e.g. codes of CIM10 and CCAM) to calculate new variables and more easily identify patients or stages of care. For example, we developed a feature “extracorporeal circulation” by identifying all CCAM codes corresponding to all procedures for which the surgery is realized with extracorporeal circulation. All these groupings were validated by physicians specialized in the field (in this case, anesthesia for cardiac and vascular surgery).

### Technologies

All databases (i.e. mirroring databases, staging area, data warehouse, datamarts) were stored on Oracle 11G (Oracle, Santa Clara, United States). The ETL process was conducted with OpenText Integration Center (OTIC, Open Text Corporation, Waterloo, Canada). Extraction for cohort creation were realized in SQL and statistical analyses were performed in R or SAS (SAS Institute, Cary, United States). OTIC offers 4 modules for (i) administration (i.e. creation and project management), (ii) ETL development, (iii) scheduling of automated loads of the data warehouse, and (iv) load execution reporting, with detailed logs of all programs (i.e. execution time, Oracle error, number of records loaded).

We set up a development environment and a production environment. Each environment contains a database server and an OTIC server. The development environment contains an extract of the AIMS database and is used to develop new loading flows. The production environment queries a complete copy of the AIMS database and stores the data warehouse with full history. In each environment, one Oracle schema is dedicated to the data warehouse, and another schema is dedicated to the data stores. In the production environment, the data warehouse is 465 Go, and the data marts is 126 Go.

### ETL process

The ETL process consists of 5 loading flows which are described in Table 1 and the following paragraphs. AIMS and PMSI data were loaded in the data warehouse with history since 2010, through an automated ETL process, executed each week (flows 1 and 2). In a second step, the data warehouse is supplemented once a year with features of high-level of information (flow 3). Then, row-oriented records of the data warehouse supply the datamarts after a pivot operation to offer wide tables with column-oriented features (flow 4). At the end of the chain, datamarts are queried on request to produce data tables for statistical analyses or dashboards (flow 5).

**Table 1:** Loading flows of the data warehouse

Flow	Data	Updating possibility in the software	Volume	Target database	Loading frequency	Period	Duration
1	AIMS: Patient history, Operating room	+	−	Data warehouse	Every week	1 year	6 h
	PMSI: Hospital stays, Unit stays, Diagnoses, Procedures						
2	AIMS: Measurements, drug administrations, events of the anesthesia procedure	−	+	Data warehouse	Every week	2 sliding weeks	1 h
3	Features	−	+	Data warehouse	Once a year	1 year	3 h
4	Pivot tables	NA	+	Datamarts	Once a year	1 year	
5	Cohorts	NA	−	Datamarts	On demand	Depending on the study	

*AIMS* Anesthesia Information Management System, *PMSI* hospital discharge reports, *NA* not applicable

#### Step 1: mirroring

As a prerequisite, we therefore had to work from copies of the production databases, in order not to slow down the performance of the software. In addition to these copies, we have set up a staging area where data are cleaned, transformed and combined between sources.

#### Step 2: raw data extraction

Software databases include dozens of tables to store clinical data, but also software configuration elements, terminologies and logs. This step aims to discover the structures of the AIMS and PMSI databases, and to identify the relevant tables and columns that were extracted from operational databases and provided to the staging area for further transformations.

For this step, we relied on experts in the field to understand how the records were documented, and what they reflected about patient care. Anesthetists and nurse anesthetists opened files individually to control records, and verify the concordance between the records in the database and the actual situation of the care.

#### Step 3: transform

The transformation stage was the most challenging one as it aimed at transforming raw and heterogeneous data into a common and homogeneous data model. For this purpose, this step involved several operations: the record linkage between sources (i.e. linking the anesthesia record from the AIMS to the patient file from the PMSI), the mapping of local vocabulary to standardized concepts, the transcoding of modalities, the filtering of erroneous records, and the homogenization of the data structure.

During this step, several data inconsistencies were addressed. First, missing or erroneous patient and stay identifiers were corrected. Secondly, the measurement and drug units were conversed to standard units (e.g. gram to milligram when both units are used for the same drug). Last, measurements and drugs administrations were flagged with a quality indicator documenting whether the value is expressed with a correct unit and whether the value is in the range of expected values. This indicator was useful to know which records to take into account in order to calculate features.

#### Step 4: load

A first part of the loading process is executed automatically every week.

Master data are integrated in the dimension tables of the data warehouse in a first step of the loading process. An initial load was initiated at the start of the project, and these tables can be completed when new concepts appear in the applications (e.g. new parameters monitored in the operating room).

Transactional data are loaded in the data warehouse following the master data, through two flows that are executed automatically every week. A first flow is related to low volume data that may have been updated in the AIMS (mainly patient history). The flow covers one year of background information and takes 6 h (flow 1). A second flow involves larger data that cannot be updated (i.e. automatically recorded measurements). This flow covers two sliding weeks and requires 1 h (flow 2). The two flows overwrite existing data. Due to the volume of data and the loading time, we have chosen an incremental updating rather than a full reload [19].

#### Step 5: feature extraction

In addition to those latter collected data, new data were computed in a second pipeline to facilitate the data reuse for research purposes, and in particular to get rid of the dependence on time (flow 3). First, periods of interest were set from temporal events. They can define perioperative periods or postoperative periods, for generally the explanatory and response variables. Secondly, during those periods, we derived features from the perioperative and postoperative measurements and events, and specified indicators such as the count, minimum, maximum, median or mean values, as well as more complex values such as hypotension, tachycardia, oxygen desaturation [7, 20]. The features were built from expert rules and have often required back and forth between physicians and data scientists. The duration of feature extraction depends on the features involved and can take from a few minutes to several hours.

#### Step 6: pivoting and data mart constitution

At the end of ETL process and feature extraction, the data warehouse is constituted of tables organized in rows, with one row per record. These records are data collected by the source software (e.g. drug administrations, procedures) or secondarily computed features. This organization is adapted for the loading of the data warehouse (with transactional or batch queries) but is not suitable for cohort creation because of the multidimensionality of the tables. Therefore, a pivot operation transforms row-oriented records into wide tables with one row per statistical unit and one feature per column (flow 4). As a result, the constitution of a data table to perform statistical analysis or data visualization will only consist in the application of filter and variable selection criteria. The pivot operation takes 5 min per final table of the datamarts.

#### Step 7: cohort creation

The last step consists of building a list of patient, stay, or procedure identifiers that meet inclusion criteria, and selecting relevant variables (flow 5). This step is usually performed using a SQL query that only requires joins between relevant tables, thanks to the work previously done in pivot. The query only takes a few minutes. If necessary, additional data manually collected are added to the extraction. This step may also includes an occasional manual check of some records.

### Data quality and monitoring of the ETL process

Data quality and ETL process were controlled and monitored by several methods.

First, we implemented data quality checks to assess completeness, correctness, concordance and plausibility of records [21]. These checks were implemented with SQL queries and were related to the presence of values, the detection of abnormal values (e.g. heart rate measurements outside the acceptable range), or the assessment of concordance between records (e.g. operating room date included during the hospital stay dates). They were first applied to the source databases, and then to the data warehouse after loading. Whenever necessary, a feedback to the anesthetists allowed to verify a record, and its concordance with the care.

Secondly, based on inconsistencies and missing data found earlier, we defined a set of rules to improve the completeness of key events such as surgery and anesthesia steps [22].

Last, the ETL process is fully monitored by OTIC. It records the environment context of the ETL and the events occurring during the process, such as database errors, process stops and runtimes. A complete dated log allows to identify which part of the program is involved when there is a failure. Therefore, the processes can be corrected and restarted when necessary.

### Ethics

Each extraction of data for a study is subject to a declaration to the Commission nationale de l'informatique et des libertés (CNIL, the National Commission on Informatics and Liberty), an independent French administrative regulatory body whose mission is to ensure that data privacy law is applied to the collection, storage, and use of personal data. Each extraction must also be reported to the Data Protection Officer of the Lille University Hospital.

### Usage

We set up a process to structure the operation of the data warehouse for research purposes [23]. It is organized around three main meetings between physicians, computer scientists, and statisticians. The data scientist acts as a coordinator, leads meetings, and checks each milestone. The aim of the first meeting is to decide the primary and secondary objectives of the study. The aim of the second meeting is to validate the statistical protocol. The data is then extracted and the statistical analyses are performed. Finally, the results are presented, explained and discussed during the third and last meeting. Additional meetings could be set up in case of difficulties during data extraction, and if there is a need to manually verify extractions.

We also set up clinical dashboards dealing with anesthesia unit management and quality assessment [24]. User needs and technical requirements were identified in end-user interviews and then synthesized. Several representations were then developed and submitted to end-users for appraisal. Lastly, dashboards were implemented and made accessible via the hospital network. Seventeen themes were identified and ten dashboards were finally implemented and deployed. The dashboards covered various aspects of overall unit activity, compliance with intraoperative hemodynamic guidelines, ventilation and monitoring, and documentation of the anesthesia procedure.

## Summary after 10 years

It required the involvement of one full-time person (the data scientist), along with help from the anesthesiologists to understand the clinical domain, and how AIMS was used. Two experienced computer scientists also provided expertise in ETL development and data warehouse modeling.

All anesthesia procedures occuring after the 2010-01-01 were loaded into the data warehouse. Between 2010 and 2020, 636 784 anesthesia records have been implemented, for 353 152 patients. The Table 2 described the number of records per table.

**Table 2:** Number of records per entity

Table	Number of records
Patient	353 152
Intervention	636 784
Measurement	3 798 604 450
Events and drugs	151 320 999
Hospital stay	575 310
Unit stay	863 700
Procedure	5 354 754
Diagnosis	3 883 340

The data warehouse was deployed at the same time in all departments of the University Hospital. New features appeared over time depending on the studies that were conducted, and when a feature was developed specifically for a study, it was then made available to the whole perimeter of the data warehouse.

We presented the progress of the project throughout the last ten years at care unit and department meetings, and during thematic days (e.g. Regional Congress of Anesthesiology). The auditors were anesthesiologists, residents, and nurse anesthetists. The presentations included some technical points on the implementation of the data warehouse, but especially illustrations on its potential uses. We also emphasized the importance of properly documenting the procedure in the AIMS to have usable data.

We conducted about 15 studies per year, in the perspective of medical theses. Several of these works have also been published. In addition to the standard time need for submission, review and editing of the manuscript, retrospective studies requires several weeks of work when the variables are simple and already available, to several months when it is necessary to develop new features. The time saving, when the development of the warehouse is well advanced, is the fact of reusing features already available in the data marts.

A first retrospective study compared hydroxyethyl starch (HES) and non-HES patients using a propensity score matching [25]. Postoperative acute kidney injury (AKI), defined by stage 3 of the Kidney Disease Improving Global Outcomes score, was the primary outcome. The intraoperative use of moderate doses of 6% HES 130/0.4 was not associated with increased risk of AKI. In a second retrospective study, we included adult patients who underwent fenestrated/branched endovascular aortic repairs between October 2010 and December 2018 [26]. Myocardial injury after non-cardiac surgery (MINS) was defined as a high sensitivity troponin (HsTnT) level ≥ 14 ng/L (MINS14) or ≥ 20 ng/L (MINS20). Of the 387 included patients, 240 (62.0%) had MINS14 and 166 (42.9%) had MINS20. Both conditions were significantly associated with poor 2-year survival. Age, revised cardiac risk index, duration of surgery, pre-operative estimated glomerular filtration rate, and haemoglobin level were identified as independent predictors of MINS. In a third study, we formed two groups: (i) an anaphylaxis group of 49 patients with tryptase/histamine assay data and allergy workup, (ii) a control (hypotension) group of 555 patients with severe hypotension (i.e. mean arterial pressure less than 50 mmHg) with a cause other than anaphylaxis after anesthesia induction [27]. The minimum ETco2 value was significantly lower in the anaphylaxis group than in the hypotension group. In multivariable analysis, minimum ETco2 was associated with anaphylaxis after adjusting for confounders and competing predictors, including arterial pressure, heart rate, and peak airway pressure.

## Lessons learnt

**Fig. 3:**
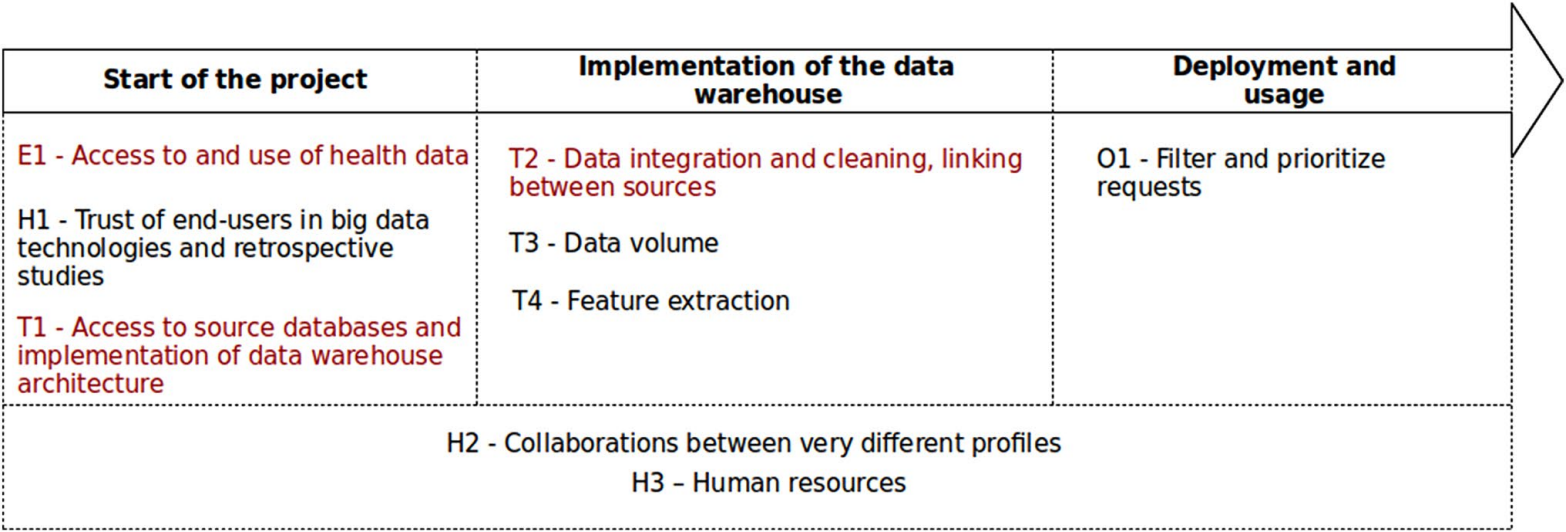
Challenges encountered over the course of a data warehouse project. Red items represent challenges that will stop the project if they are not fully mastered

We have identified a set of barriers on several aspects: ethical and legislative, technical, human and organizational. These barriers and the related proposals to handle them are described in Table 3 and in the following sections. The Fig. 3 arranges these challenges in a temporal manner according to the main steps of a data warehouse project.

**Table 3:** Barriers encountered during the project

Aspect	Barriers	Tips—Solution
Ethical	E1—access to and use of health data	Tip 7
Human	H1—trust of end-users in big data technologies and retrospective studies	Tip 2, Tip 3
Human	H2—collaborations between very different profiles	Tip 6
Human	H3—human resources	Tip 8
Technique	T1—access to source databases and implementation of data warehouse architecture	Tip 1
Technical	T2—data integration and cleaning, linking between sources	Tip 5
Technical	T3—data volume	Tip 4
Technical	T4—feature extraction	Tip 6
Organisationel	O1—filter and prioritize requests	Tip 7, Tip 8

### Ethical and legislative barriers

The access to and use of health data is regulated and subject to strict practices (E1). Legislation on the reuse of health data has evolved over the past few years. At the beginning of the project, the database had to be declared to the Data Protection Officer of the Hospital and to the CNIL, and each study had to be validated by an ethical committee. At the European level, legislation has evolved with the adoption of the General Data Protection Regulation in 2016. This regulation aims to strengthen both the protection of persons concerned by the processing of their personal data and the responsibility of those involved in such processing. Main requirements imply (i) the explicit and positive consent of the patient to the re-use of their data, (ii) the right to erasure, and (iii) the Privacy Impact Assessment.

### Technical barriers

The first technical barrier (T1) relates to the access to the source databases and the implementation of the data warehouse architecture, and in particular, the creation of a workspace to transform raw data.

The second technical barrier (T2) concerns data quality and the data transformation stage of the ETL process. AIMS and PMSI suffer from a wide range of data quality problems that come from manual inputs, software settings that evolve over time, different data entry practices by person and department, monitors not connected to the software, etc. Moreover, crucial data may be missing and complicate the linking of multiple data sources. Last, data cleansing operations that have been put in place, and loading in its entirety, are complicated to evaluate and track because they involve millions of rows that cannot be checked manually.

Data volumetry (T3) is a key element in the processing of monitoring data, with several measurements per minute for the same signal during the few hours of the intervention. As a result, the execution time of the ETL steps related to the measurements quickly becomes exponential when the volume increases.

Even when the data have been cleaned and are well organized in the data warehouse, their multi-dimensional and time-dependent aspects need transformations to obtain information directly usable for analysis. The step of feature extraction is crucial and requires knowledge of the domain (T4).

### Human challenges

Data warehousing and data reuse are new technologies in hospitals, separate from clinical routine and different from prospective clinical research expertise. Concerns, questions, and misunderstandings on the part of clinicians, researchers, and institutions have been numerous regarding the implementation and use of this new tool (H1). The main concern expressed at the beginning of the project was being watched and evaluated individually. When in use, there have been concerns about the reuse of automatically collected data and the level of evidence of retrospective studies, in contrast to prospective clinical studies where data are specifically collected. This type of project brings together very diverse profiles (computer scientists, clinicians, researchers, lawyers, researchers, statisticiens, managers) and it is not always easy for non-physicians to have their opinions and expertise heard, even in an IT project (H2).

Last, we encoutered financial difficulty to perpetuate positions on this project and to benefit from the availability of anesthesiologists' time, who are already understaffed in clinical practice (H3).

### Organizational challenges

The availability of the data warehouse has provided opportunities to conduct research at lower cost (compared to prospective research) and as a result it has led to being overwhelmed by dozens of requests each year.

### Tips to address barriers and challenges

**Fig. 4:**
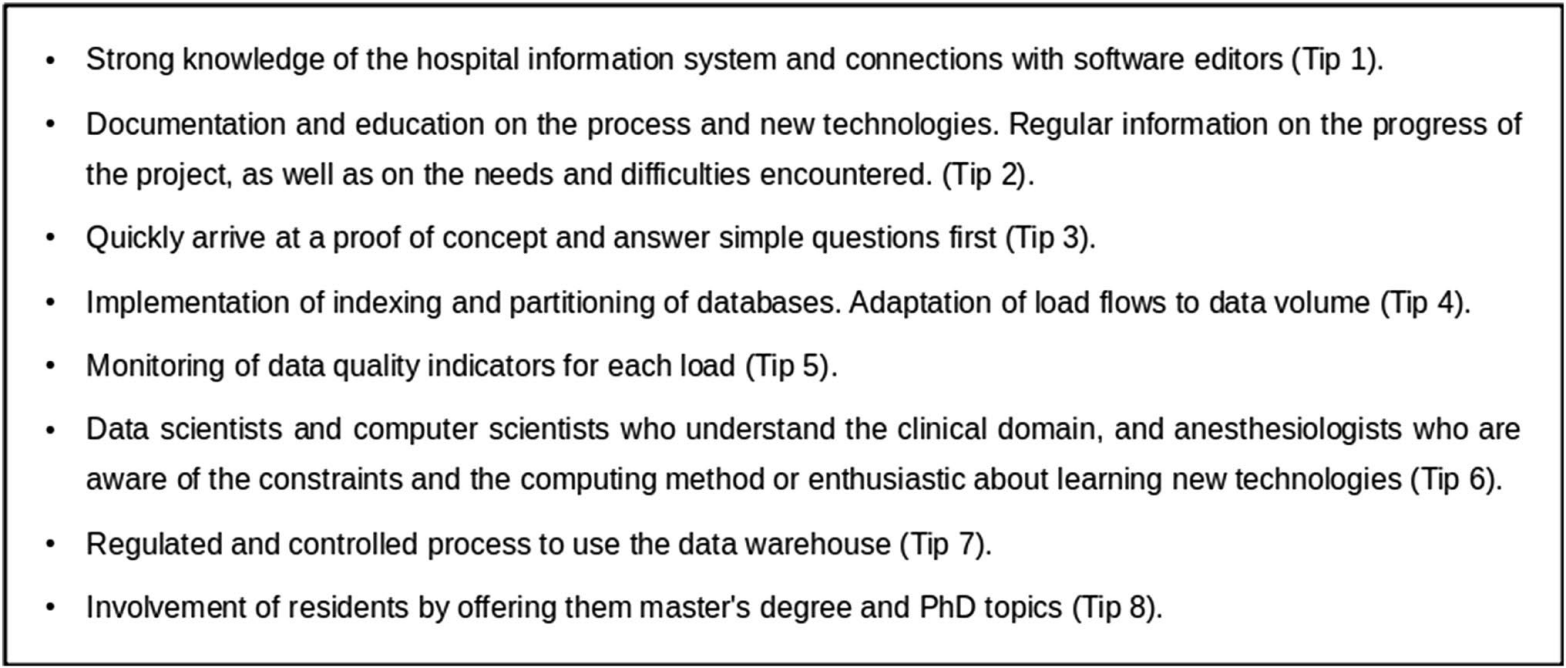
Tips for setting up and managing a data warehouse project

We propose 8 tips to manage the barriers previously listed. They are decribed below and resumed in Fig. 4.

Collaboration with the IT department to benefit from their strong knowledge of the hospital information system and to ensure that the architecture of the data warehouse was compliant with their requirements. We also had connections with software editors to understand the structure of the databases linked to their software. (Tip 1).

Documentation and illustration of the data warehouse to educate on this new tool. Inform regularly on the progress of the project (e.g. what data is available), as well as the needs and difficulties encountered. Disseminate the use that is made of the data warehouse and the studies that have been completed. (Tip 2).

Quickly arrive at a proof of concept and answer simple questions first with only a few variables, from demographic data (e.g. sex, age, ASA physical status) or activity (e.g. number of anesthesia procedures per period). Very simple dashboards that allow to have a return on the activity. (Tip 3).

Avoid exponential execution time due to volume by setting up indexing, partitioning, and in adapting load flows to the volume of data. The goal is to get rid of the growing volume of data, and to work on a constant volume of data. (Tip 4).

Because of the high volume of data, it is not possible to check the quality of individual records. We computed and monitored data quality indicators at each load of the data warehouse. These indicators covered the number of records loaded, the compliance with the value ranges and the documentation of essential elements of the anesthesia procedure. (Tip 5).

Involve data scientists and computer scientists who understand the clinical related domain, and on the counter side, anesthesiologists who are aware of the computing method or are enthusiastic about learning new technologies (Tip 6).

We have set up a regulated and controlled process to use the data warehouse. It guides users from the definition of the research question to the interpretation of the results [21]. (Tip 7).

Residents who wished to pursue an academic career were involved in the project by offering them master's degree topics and PhD related to big data (Tip 8).

## Current developments

### Implementation of a common data model

As it stood, the architecture of our data warehouse was suitable for local use only, because of a data format and vocabulary that were based on our software or the configuration that we had set up in Lille. The methods and tools we had developed could hardly be shared with other centers and therefore, recently, we have transformed our data to implement them into the Observational Medical Outcomes Partnership (OMOP) common data model (CDM) [28]. The OMOP-CDM provides standardized table and column names, as well as a unified vocabulary [29]. A set of tools and methods are provided and shared with the model, and we were also able to share some of our work [30]. OMOP CDM continues the Inmon architecture, with a warehouse as a the unified data source. It continues the Inmon architecture, the data marts are only constituted in a second step, to build the data tables for each cohort.

To align with the OMOP model, the passage in the operating room was considered as a VISIT_DETAIL record, in the same way as a stay in the intensive care unit or a passage to the emergency room. All other events and data resulting from the OR, i.e. drug administrations, anesthesia and surgery steps, measurements, have been placed in the appropriate tables, i.e. DRUG_EXPOSURE, PROCEDURE_OCCURRENCE, OBSERVATION and MEASUREMENT. The link between these records and the passage in the operating room was maintained through the foreign key column VISIT_DETAIL_ID in each of these tables. Local vocabularies were mapped to the standard vocabularies recommended by the OMOP community, i.e. SNOMED (Systemized Nomenclature for Medicine), UCUM (Unified Code for Units of Measure), LOINC (Logical Observation Identifiers Names and Codes), RxNorm, ICD10. We provided support to implement the OMOP CDM with anesthesia data in providing a list of required vocabulary, the transformations between AIMS structure and OMOP CDM, a set of queries and 4 clinical dashboards [31].

### Federated learning

In order to gather sufficiently large data sets, it is often necessary to set up multicenter studies; yet, centralization of records can be difficult, either for practical, legal or ethical reasons. As an alternative, federated learning enables leveraging multiple centers' data without actually collating them [32]. The data warehouse is an excellent tool to promote federated learning, especially if the data warehouse model is the same in the different centers. In this case, the cohort creation queries can also be shared, upstream of the statistical analysis scripts.

We performed a proof of concept with a dataset of caesarean sections under spinal anaesthesia clinical we split among 4 hypothetical centers and tested an algorithm for logistic regression, including confidence intervals computation [33]. This showed that the results were identical to the centralized version and that the confidence intervals were tighter than for each individual center. After this in vitro proof of concept, we are now trying to implement federating learning in several institutions [34].

## Discussion

In this article, we reported the development, implementation and use of an anesthesia data warehouse in a French university hospital. We highlighted the difficulties encountered during the conduct of this project, and we tried to provide advice to handle them.

The development of our data warehouse started 10 years ago and lasted 4 years. It is loaded on a weekly basis and currently includes data related to more than 600 000 anesthesia procedures. It covers all events occurring in the operating room, as well as the hospital stay and the related diagnoses and medical procedures. We also integrated biology results, transfusions, a mortality database, and manually collected data. In addition to the raw data, it was necessary to extract features in an automated way, generally by applying rules defined by domain experts. The data warehouse has permitted us to perform one hundred and twenty retrospective studies for resident works, and for some publications [25–27].

The difficulties often put forward with this kind of project are mainly technical, and are linked to the volume of data and poor data quality, which generally result from manual entries or lack of documentation. However, the fact remains that the human and organizational aspects are often the most difficult to overcome. The implementation of such a project requires the articulation of several disciplines and skills.

At the start of the project, we implemented our own data model. Now, we have moved to the OMOP CDM to be able to collaborate more easily with other centers, and share our tools and methods.

The next step of the project is to share our experience and tools to help other centers for developing their own data warehouse and implementing the OMOP CDM. We are currently collaborating in this sense with several French university hospitals. Another area for improvement in the project is the implementation of federated learning in routine, in order to conduct multicenter studies. We are also currently working on providing open access to our data warehouse, on the same way as the MIMIC project and the open-access databases of Physionet [35]. This is a real challenge in regard to local and European laws. Last, the data warehouse must also provide physicians with feedback on their activity, and assess the compliance with clinical guidelines resulting from clinical research.
